# Repurposing the antispasmodic drug pinaverium bromide as a novel antifungal agent and synergist against *Candida albicans*

**DOI:** 10.1080/21505594.2026.2682573

**Published:** 2026-06-01

**Authors:** Jing Yao, Zhiyu Yang, Xudong Hang, Tianyu Chen, Binglei Li, Ting Shi, Keao Quan, Jingchen Xu, Liping Zeng, Ganzhu Feng, Hongkai Bi

**Affiliations:** a NHC Key Laboratory of Tropical Disease Control, School of Life Sciences and Medical Technology, Hainan Medical University, Haikou, Hainan, China; b Department of Respiratory and Critical Care Medicine, The Second Affiliated Hospital of Nanjing Medical University, Nanjing, Jiangsu, China; c Department of Pathogen Biology, Jiangsu Key Laboratory of Pathogen Biology, Nanjing Medical University, Nanjing, Jiangsu, China; d Department of Respiratory and Critical Care Medicine, The Affiliated Taizhou People’s Hospital of Nanjing Medical University, Taizhou, Jiangsu, China

**Keywords:** pinaverium bromide, Candida albicans, synergistic action, drug repurposing, amphotericin B

## Abstract

Fungal infections represent a significant and growing threat to public health, exacerbated by an expanding population of immunocompromised individuals and the increasing prevalence of resistance to conventional antifungal agents. Drug repurposing offers a strategic and efficient pathway for antifungal discovery, leveraging existing pharmacotherapies to reduce development costs and mitigate safety risks. This study evaluated the antifungal potential of pinaverium bromide, an FDA-approved antispasmodic drug for functional gastrointestinal disorders, against the prevalent pathogen Candida albicans. Our in vitro analyses revealed that pinaverium bromide demonstrated standalone antifungal activity and acted synergistically with amphotericin B or azole drugs. Moreover, it effectively attenuated key virulence factors of C. albicans, including hyphal formation and biofilm development. The therapeutic efficacy of both monotherapy and combination therapy with amphotericin B or voriconazole was validated in two murine models of systemic candidiasis. Mechanistically, pinaverium bromide disrupted mitochondrial function, induced apoptotic cell death, and impaired iron homeostasis in C. albicans. When combined with amphotericin B, it potentiated the drug’s effect by amplifying reactive oxygen species accumulation and enhancing membrane permeabilization. These findings support the potential of pinaverium bromide as a novel antifungal agent, either when used alone or in combination with established antifungal therapies.

## Introduction

Fungal infections pose a significant global health burden, accounting for approximately 1.5 million deaths annually [1]. Among fungal pathogens, *Candida* species are the predominant causative agents of fungal infections in humans, with *Candida albicans* being the leading culprit of opportunistic infections [2]. While typically a commensal colonizer of mucosal surfaces (oral, gastrointestinal, genital), *C. albicans* can become pathogenic, particularly in immunocompromised hosts [2,3]. This pathogen causes both mucosal and systemic infections; notably, systemic candidiasis carries a mortality rate of 30–40% despite current antifungal therapies [2,4]. A key virulence determinant is the morphological transition from yeast to hyphae, which facilitates host cell adhesion, invasion, and damage [5]. Hyphal formation also plays a pivotal role in *C. albicans* biofilm development on host tissues or medical devices (e.g. catheters and implants). These biofilms confer resistance to host immune defenses and antifungal agents, leading to persistent, recurrent infections and drug resistance [6].

The primary classes of commonly used antifungal agents, including polyenes, azoles, and echinocandins, exhibit limitations. Amphotericin B (AMB), the most widely utilized polyene with potent antifungal activity against a broad range of pathogenic fungi, is employed to treat systemic fungal infections. Nevertheless, it causes dose-dependent nephrotoxicity in the host [1]. Azoles, such as voriconazole (VRC), itraconazole (ITR), and fluconazole (FLC), are frequently compromised by pharmacokinetic interactions, emerging resistance, and host toxicity [7]. Even echinocandins, recommended as first-line therapy for invasive candidiasis, have also developed resistance in recent years [1,8]. Concurrently, the population at risk for fungal infections is expanding due to medical advances and immunomodulatory therapies. This convergence of factors, including rising disease incidence, constrained antifungal pipeline, and escalating resistance, underscores an urgent need for novel therapeutic strategies.

Developing new antifungals is inherently challenging because *C. albicans*, as a eukaryote, shares numerous conserved functional proteins with humans, limiting target selectivity. In this context, drug repurposing emerges as a viable strategic alternative. This approach capitalizes on existing drugs, offering the potential to streamline development, reduce costs, and circumvent the significant safety uncertainties of traditional discovery pathways [9]. However, thorough investigation of the underlying pharmacological mechanisms for repurposed agents remains crucial [10]. Combination therapy represents another vital approach, enhancing the efficacy of conventional agents (e.g. azoles) through synergy effects [11]. This strategy can lower effective dosages, reduce toxicity, overcome existing resistance, and impede the evolution of new resistance mechanisms by combining distinct modes of action and reducing pathogen burden. Consequently, drug combinations are considered a promising strategy for combating resistant fungi and extending the utility of current antifungals [12].

In this study, we identified an FDA-approved drug, pinaverium bromide (PB), with antifungal activity against *C. albicans* through phenotypic screening of MedChemExpress (MCE) Drug library. We further characterized its synergistic interactions with AMB and azoles *in vitro* and validated the efficacy of both PB monotherapy and combination therapy in murine models of systemic candidiasis. Mechanistic investigations revealed that PB potentiates AMB activity by augmenting intracellular reactive oxygen species (ROS) accumulation and increasing membrane permeability. These findings highlight the therapeutic potential of PB for the treatment of invasive candidiasis.

## Materials and methods

### Strains, culture conditions and reagents

*C. albicans* SC5314, ATCC10231 and ATCC14053 were purchased from the American Type Culture Collection (ATCC; Manassas, VA, USA), while 14 clinical isolates (listed in Table 1) were obtained from urine, blood or the oral cavity samples at the Stomatological Hospital of Jiangsu Province in Nanjing, China. Other non-*albicans Candida* species (Table 1) used in this study were obtained from the laboratory stocks. All *Candida* strains were grown on Sabouraud dextrose agar (SDA) plates and cultured in RPMI-1640 (Gibco; Grand Island, NY, USA) supplemented with morpholinepropanesulfonic acid (MOPS; Macklin, Shanghai, China) at 35°C. PB was purchased from MedChemExpress (MCE; Monmouth Junction, NJ, USA), and L-AMB from New Aisa Pharma (Shanghai, China). AMB and FLC were obtained from Aladdin Industrial Corporation (Shanghai, China), and VRC and ITR from Tokyo Chemical Industry (Shanghai, China). All compounds were dissolved in dimethyl sulfoxide (DMSO) and stored at −80°C.

**Table 1.** MIC of PB against fungal strains (μg/mL).

Strains	Antifungal drugs^b^				
	PB	AMB	VRC	ITR	FLC
*C. albicans* SC5314	16	2	0.008	0.25	0.25
*C. albicans* ATCC10231	16	2	0.5	2	1
*C. albicans* ATCC14053	16	1	0.25	4	8
*C. albicans* C2 ^a^	16	2	64	32	16
*C. albicans* C3 ^a^	16	2	64	16	16
*C. albicans* C4 ^a^	16	2	2	16	8
*C. albicans* C5 ^a^	16	2	256	>128	>128
*C. albicans* YY-1–4 ^a^	16	2	4	8	32
*C. albicans* YY-21–08 ^a^	16	2	1	16	16
*C. albicans* CANT1 ^a^	16	2	0.25	1	16
*C. albicans* CANT2 ^a^	16	1	16	8	16
*C. albicans* CANT3 ^a^	8	2	0.5	2	8
*C. albicans* CANT4 ^a^	16	2	1	0.25	8
*C. albicans* CANT5 ^a^	8	2	1	8	8
*C. albicans* BHKS681 ^a^	16	1	64	>128	>128
*C. albicans* BHKS682 ^a^	16	1	256	>128	>128
*C. albicans* BHKS683 ^a^	16	1	64	>128	>128
*C. tropicalis* GDMCC2.147	8	2	0.031	>128	1
*C. parapsilosis* ATCC22019	8	2	0.031	64	2
*C. glabrata* ATCC36583	32	1	0.25	64	16
*C. lusitaniae* GDM2.145	8	2	0.031	64	4
*C. krusei* ATCC6258	32	2	0.25	64	32
*C. auris* DSM21092	32	2	0.031	2	2
*C. auris* CBS12373	32	2	2	4	128

^a^Clinical isolates. ^b^ PB: pinaverium bromide; AMB: amphotericin B; VRC: voriconazole; ITC: itraconazole; FLC: fluconazole. —, not tested.

### Chemical screening

A chemical screen was conducted using a library of 393 FDA-approved compounds obtained from MedChemExpress (MCE; complete list in Table S1). The assay was performed against the voriconazole-resistant *C. albicans* clinical isolate, C5. A suspension of the isolate was prepared in RPMI-1640 medium to a final density of approximately 5 × 10^2^ CFU/mL. Aliquots of this suspension were dispensed into 96-well plates and treated with each compound at a final concentration of 25 µM in a total volume of 100 µL per well. Plates were incubated statically at 35°C in the dark for 24 h, after which growth was assessed visually. Compounds producing clear or slightly hazy wells, indicating growth inhibition relative to untreated controls, were selected as preliminary hits for further analysis. The entire screening procedure was performed in three independent biological replicates to ensure robustness.

### Antifungal susceptibility testing

The MIC values were determined using broth microdilution assays in compliance with Clinical and Laboratory Standards Institute (CLSI) guidelines reference document M27-A4 (CLSI 2017). For *Candida* spp., twofold serial dilutions of each compound were prepared in 96-well plates containing 90 µL RPMI-1640 medium per well. A 10 µL inoculum, standardized to yield a final cell density of approximately 5 × 10^2^ CFU/mL, was added to each well. Plates were incubated at 35°C for 24 h. The MIC was defined as the lowest compound concentration that completely inhibited visible growth. Standard clinical antimicrobial agents (AMB, VRC, ITR and FLC) were included as comparators in all assays. All MIC determinations were performed with three independent biological replicates.

### Checkerboard assay

Synergy analyses of PB with AMB, VRC, ITR, or FLC were performed using a checkerboard titration method, as described previously [13]. Briefly, the experiments were carried out on 96-well plates at a cell density of 5 × 10^2^ CFU/mL and each well contained a final volume of 100 µL RPMI-1640 medium. The concentration ranges for each drug were selected based on the MIC of the tested strain (PB, 0.5–128 μg/mL; AMB, 0.063–4 μg/mL; VRC, 0.001–256 μg/mL; ITR, 0.016–128 μg/mL; FLC, 0.016–128 μg/mL). After incubation at 35°C for 24 h, the MIC value of each drug was determined as described above. The synergistic effect was estimated by FICI and calculated as follows: FICI = (MIC of drug A in combination)/(MIC of drug A alone) + (MIC of drug B in combination)/(MIC of drug B alone). A FICI of 0.5 or less was defined as synergism, >0.5 to 1 as addition, >1 to 4.0 as indifference, and >4 as antagonism [14].

### Time-killing assay

The killing assays were conducted in 96-well microplates following exposure to various concentrations of PB. *C. albicans* SC5314 cells were diluted to a density of approximately 2 × 10^5^ CFU/mL in YPD medium supplemented with 1×, 2×, 4×, 8 ×, 16×, or 32× the MIC of PB. The cell suspensions were incubated at 35°C with shaking (200 r/min). Then, 100 μL of the suspension was collected from each sample at the indicated time points (0 h, 4 h, 8 h, 12 h and 24 h), and serial dilutions were plated on SDA plates to quantify the viable cells (expressed as CFU/mL). All measurements were carried out with three independent biological replicates.

### Resistance development assay

The resistance development assay was performed by treating *C. albicans* SC5314 repeatedly with PB, AMB, or VRC in RMPI-1640 medium as described previously with minor modifications [15]. Briefly, MIC testing was first conducted for tested agents, as described earlier. At the end of incubation (35°C for 24 h), the fungal cells growing in the well with the half-MIC concentration were harvested and adjusted to an OD_625_ of 0.08 to 0.1. After a 1:1,000 dilution with the RMPI-1640 medium, the inoculum was subjected to the next passage MIC testing, and the process was repeated for 60 passages.

### Hyphal induction assay

The effect of PB on the hyphal development of *C. albicans* was evaluated by quantifying filamentous growth, as described previously [16]. Briefly, *C. albicans* SC5314 cells were inoculated at a density of 1 × 10^5^ CFU/mL in RPMI-1640, Lee’s, or Spider medium containing various concentrations of PB (4, 8, 16, and 32 μg/mL) or an equivalent volume of DMSO as a control and incubated at 35°C for 4 h. Hyphal morphology was subsequently examined using an Axio Observer 5 inverted light microscope (Carl Zeiss; Oberkochen, Germany). The presented data are representative of two independent experimental replicates.

### Measurement of [Ca^2+^]_i_

The [Ca^2+^]_i_ in *C. albicans* SC5314 following PB treatment was measured using the fluorescent probe Fluo-4/AM (MCE; USA), a cell-permeable Ca^2+^ indicator [17]. Briefly, yeast cells were activated, harvested, and washed three times with D-Hanks’ buffer (Biosharp; Beijing, China). The cells were then resuspended to an optical density at 530 nm (OD_5__30_) of 0.2 (approximately 10^6^ CFU/mL) and then loaded with 2.5 μM Fluo-4/AM for 45 min at 35°C in the dark. After staining, the cells were washed three times with D-Hanks’ buffer via centrifugation and concentrated 10-fold. The OD_5__30_ was re-measured after this concentration step. Aliquots of the cell suspension were treated with PB (32 μg/mL), chelerythrine (1 μg/mL), or an equivalent volume of DMSO as a control. Chelerythrine was used as a positive control, as it is known to increase the [Ca^2+^]_i_ in *C. albicans* [18]. Fluorescence was immediately monitored for 200 min at 40-min intervals using a microplate reader (BioTek; VT, USA) with excitation/emission wavelengths of 485/526 nm.

### Antibiofilm assay

Biofilm formation was performed in 96-well microtiter plates according to a previously described method, with minor modifications [19]. Briefly, *C. albicans* SC5314 cells were diluted with RPMI-1640 medium to a concentration of approximately 1 × 10^6^ CFU/mL. A 100-µL aliquot of the cell suspension was then dispensed to each well. After incubation at 35°C for 2 h, the medium was gently aspirated, and non-adherent cells were removed by washing twice with sterile PBS. Subsequently, each well was supplemented with 100 µL of RPMI-1640 medium, either with or without various concentrations of PB (16, 32, 64, or 128 μg/mL). The microtiter plates were then incubated at 35°C for 48 h. AMB was used as a control. The total biomass of the biofilms was quantified using crystal violet staining method [20]. The inhibition of biofilm formation was expressed as a percentage of the remaining biofilm biomass relative to that of the untreated ones. All experiments were performed in three independent biological replicates.

To evaluate the ability of PB to eradicate preformed biofilms, *C. albicans* SC5314 cells were cultured for 48 h, and the medium was aspirated and then washed with PBS twice. Fresh RPMI medium containing various concentrations of PB (16, 32, 64, or 128 μg/mL) or AMB was added to the wells for another 24 h incubation. Afterward, the Alamar Blue assay, a colorimetric assay based on the cellular reduction of resazurin to resorufin, was used for the quantification of the washed biofilms, as described previously [19]. Alamar Blue reagent (10 µl per 100 µl of medium) was added to each well followed by 2 h incubation in the dark at 35°C. Then, images were captured using a camera, and the fluorescence was detected at an excitation (Ex) wavelength of 545 nm and an emission (Em) wavelength of 590 nm with a microplate reader (Biotek, VT, USA). The percentage of surviving biofilm cells was calculated relative to the control treatment.

Cell viability within the biofilm was assessed using the fluorescent dyes, SYTO-9 and propidium iodide (PI) (Invitrogen; Carlsbad, CA, USA). The mature biofilms as described above were treated with 1 mL of RPMI-1640 medium supplemented with varying concentrations of PB (32, 64, or 128 μg/mL) at 35°C for 24 h. AMB was used as a control. Non-adherent cells were removed by washing twice with PBS. The biofilms were then stained with 200 µL of SYTO-9 (3 μM) and PI (100 μg/mL) at 35°C for 30 min in the dark. Excess dye was rinsed off with PBS. The images were observed using a CLSM (LSM710; Carl Zeiss; Oberkochen, Germany). SYTO-9 and PI were used to distinguish live (green) cells from dead (red) ones, as SYTO-9 is membrane-permeable in both live and dead cells, while PI is membrane-impermeable, staining only cells with damaged membranes.

### Transcriptomic analysis

*C. albicans* SC5314 cells suspended with 100 mL of RPMI-1640 at a cell density of 2 × 10^6^ CFU/mL were treated with 32 μg/mL PB, 1 μg/mL AMB alone or in combination at 35°C for 1 h. The fungal cells were collected and the whole sequencing process was conducted by Shanghai Personal Biotechnology Co., Ltd. (Shanghai, China). Total RNA was isolated using Trizol Reagent (Life Technologies; Fullerton, CA, USA) according to the manufacturer’s guidelines. The concentration and the integrity of RNA were determined using a NanoDrop Spectrophotometer (Thermo Scientific; Waltham, MA, USA). Three micrograms of RNA was used as the input material. Sequencing libraries were established with the TruSeq RNA Sample Preparation Kit (Illumina; San Diego, CA, USA). Briefly, poly-T oligo-attached magnetic beads were applied to mRNA purification from total RNA and fragmentation was generated with divalent cations in an Illumina proprietary fragmentation buffer at elevated temperature. First strand cDNA was synthesized via random oligonucleotides and Super Script II reverse transcriptase (Invitrogen) and subsequently second strand cDNA using DNA Polymerase I and RNase H. The remaining overhangs were transformed into blunt ends through exonuclease/polymerase activities and then these enzymes were removed. Illumina PE adapter oligonucleotides were ligated for hybridization preparation after the adenylation of the DNA fragment 3’ ends. The AMPure XP system (Beckman Coulter; Beverly, CA, USA) was employed for the purification of the library fragments, to select the preferred cDNA fragments of 400–500 bp in length. DNA fragments with ligated adaptor molecules on both ends were enriched selectively with Illumina PCR Primer Cocktail in a PCR reaction of 15 cycles. The products were purified AMPure XP reagent (Beckman Coulter) and then quantified with the Agilent high sensitivity DNA assay on a Bioanalyzer 2100 system (Agilent; Beijing, China). The cDNA library was sequenced on NovaSeq X Plus platform (Illumina; San Diego, CA, USA) by Shanghai Personal Biotechnology Co. Ltd. RNA-sequencing reads were aligned to *C. albicans* SC5314 genome from NCBI (RefSeq accession number GCF_000182965.3).

Differentially expressed genes (DEGs) were analyzed by DESeq (version 1.30.0). Paired differential gene expression with |log2 fold change|>1 and a *p* value less than 0.05 was considered to be differentially expressed. Volcano plots were charted to present DEGs with ggplots2 R package. Heat maps of DEGs were performed with the standardized fragments per kilo bases per million fragments (FPKM) values of each gene and hierarchical clustering was analyzed with Euclidean and Complete Linkage method using Pheatmap R package. Enrichment analysis of DEGs was carried out using Gene Ontology (GO) analysis with topGO. All experiments were performed in three independent biological replicates.

### Quantitative reverse transcription PCR (RT-qPCR) analysis

To validate the transcriptomic data, a subset of differentially expressed genes associated with hyphal development, biofilm formation, ROS response, and iron homeostasis was selected for RT-qPCR analysis. *C. albicans* SC5314 cells were cultured and treated under identical conditions to the transcriptomic study, and total RNA was isolated using the same methodology. Gene-specific primers were designed with NCBI Primer-BLAST, and their sequences are presented in Table S2. The *ACT1* gene was used as an internal control for normalization. Briefly, cDNA was synthesized from total RNA using the HisScript II QRT SuperMix for qPCR (+gDNA wiper) kit (Vazyme; Nanjing, China) in accordance with the manufacturer’s protocol. Subsequent qPCR amplification was performed using ChamQ SYBR qPCR Master Mix (Vazyme; Nanjing, China) on a LightCycler 96 instrument (Roche). The thermocycling conditions consisted of an initial denaturation at 95°C for 30 s, followed by 40 cycles of 95°C for 10 s and 60°C for 30 s. Gene expression levels in treated samples were quantified relative to controls using the 2^–ΔΔCt^ method [21]. All experiments were performed in three independent biological replicates.

### Measurement of intracellular ROS

Intracellular ROS levels were measured using the fluorescent dye H_2_DCFH-DA as described previously [22,23]. Briefly, *C. albicans* SC5314 cells suspended in RPMI-1640 medium at a concentration of 1 × 10^6^ CFU/mL were treated with PB (128 μg/mL) or AMB at 8×MIC, alone or in combination, at 35°C for 2 h. After incubation, the cell suspensions were centrifuged at 2,000 *×g* at room temperature for 20 min, washed twice with PBS and then resuspended in 1 mL of PBS. Subsequently, the OD_530_ was detected using a Synergy HTX multimode microplate reader (BioTek; VT, USA). Meanwhile, the suspensions were incubated with 80 μM H_2_DCFDA fluorescent probe at 35 °C for 30 min in the dark, and then the fluorescence intensities (Ex/Em = 488 nm/530 nm) were detected by a microplate reader (BioTek, VT, USA). The results presented are the fluorescence intensities normalized to cell concentration. All experiments were performed in three independent biological replicates.

### Plasma membrane potential assay

The plasma membrane potential of *C. albicans* SC5314 was assessed using the fluorescent probe DiSC_3_(5) (Mkbio; Shanghai, China). Cell suspensions were prepared in PBS at a concentration of 1 × 10^6^ CFU/mL and loaded with 1 μM DiSC_3_(5). The suspension was incubated at 35°C for 40 min in a microplate reader, with fluorescence intensity (Ex/Em = 622/670 nm) recorded at 1-min intervals. Following the loading period, the cell suspensions were treated with 10 mM KCl to establish a baseline. Subsequently, cells were exposed to PB or AMB, each at 2× MIC, either individually or in combination. Fluorescence was continuously monitored throughout a 60-min incubation at 37°C [24]. All experiments were conducted in three independent biological replicates.

### Membrane permeability assay

Membrane permeability was evaluated using propidium iodide (PI) uptake according to a previously described method [25]. PI is a high-affinity nuclear dye that enters cells with compromised membranes and exhibits red fluorescence upon binding to nucleic acids. *C. albicans* SC5314 cells were suspended in RPMI-1640 medium at a density of 1 × 10^6^ CFU/mL and treated as follows: PB alone (at 2×MIC and 8×MIC), AMB alone (at 2×MIC and 8×MIC), or a combination of both drugs (each at 2×MIC). Following a 2-h incubation at 35°C, the cell suspensions were centrifuged and the pellets were stained with 10 μg/mL PI. Staining was performed at 35°C for 30 min in the dark to prevent photobleaching. Membrane integrity was subsequently assessed by visualizing PI fluorescence using a fluorescence microscope (Carl Zeiss).

### Transmission electron microscopy (TEM) analysis

Sample preparation for TEM was performed according to a previously described method [26], with modifications. Briefly, *C. albicans* SC5314 cells were suspended in RPMI-1640 medium at a density of 5 × 10^6^ CFU/mL and treated for 8 h at 35°C under the following conditions: AMB at 2×MIC and 8×MIC, PB at 2×MIC and 8×MIC, or a combination of both drugs (each at 2×MIC). Following treatment, the fungal cells were pelleted by centrifugation at 2,000 × g for 20 min and washed twice with PBS. The pellet was then fixed with 2% glutaraldehyde in 0.1 M sodium cacodylate buffer (pH 7.4). Subsequently, the cells were embedded in 2% agarose and post-fixed in 1% osmium tetroxide overnight at room temperature. The samples were then dehydrated through a graded ethanol series, infiltrated, and embedded in Durcupan™ resin (Sigma-Aldrich; Darmstadt, Germany). Ultrathin sections (70–90 nm) were cut, mounted on grids, and stained with 2% uranyl acetate followed by lead citrate. The prepared grids were imaged using a JEM-1200 transmission electron microscope (JEOL; Akishima, Tokyo, Japan). The results presented are representative of three independent experimental replicates.

### Measurement of intracellular free Fe^2+^ production

Free Fe^2+^ level was determined by FerroOrange fluorescent probe (Dojindo Laboratories; Kumamoto, Japan), as described previously with modifications [27]. *C. albicans* SC5314 cells in RPMI-1640 medium at a concentration of 2 × 10^6^ CFU/mL were treated with varying concentrations of PB (64 or 128 μg/mL) at 35 °C for 2 h. Then, the suspension was centrifuged and washed with PBS, followed by resuspension with 300 μL of PBS. The cell suspension was applied to OD_530_ detection and stained with 1 μM FerroOrange at 35°C for 30 min in the dark, respectively. The fluorescence intensity (Ex/Em = 550 nm/600 nm) was measured with a microplate reader (BioTek; VT, USA) and normalized to the cell concentration. All experiments were conducted in three independent biological replicates.

### Molecular docking analysis

The three-dimensional structure of PB was retrieved from the PubChem database (CID: 40,703). Molecular docking simulations to investigate the interaction between PB and the transcription factor Sef1 from *C. albicans* were performed using the Schrödinger Maestro 12.8 suite (Schrödinger, LLC; New York, NY, USA). The protein structure of Sef1 was modeled using AlphaFold2 (accession: Q59UY7). This predicted structure was subsequently prepared using the Schrödinger Protein Preparation Wizard, which involved the addition of hydrogen atoms and energy minimization to refine the model. Concurrently, the ligand (PB) was prepared for docking using the LigPrep module to generate optimized low-energy conformations. Potential binding sites on the Sef1 structure were predicted using the SiteMap module. A docking grid was then generated around the primary predicted site using the Glide Grid module. Finally, flexible ligand docking was carried out using the Glide module (Standard Precision, SP) to predict the binding pose and affinity of PB within the Sef1 binding site.

### Mitochondrial membrane potential assay

The effect of PB on the mitochondrial membrane potential of *C. albicans* was evaluated using the fluorescent probe JC-1. In normal cells with high ΔΨm, JC-1 forms red-fluorescent aggregates, whereas in depolarized cells, it remains as a green-fluorescent monomer. Briefly, *C. albicans* SC5314 cells were suspended in PBS at a density of 1 × 10^6^ CFU/mL and loaded with 10 μg/mL JC-1 by incubation at 35°C for 30 min in the dark. During the loading period, fluorescence intensities were monitored at 1-min intervals using a microplate reader (BioTek) with the following excitation/emission settings: red fluorescence (aggregate form) at 550/600 nm and green fluorescence (monomeric form) at 485/535 nm. Following dye loading, the cell suspensions were treated with PB (128 μg/mL) or AMB at 8×MIC and incubated for 2 h at 35°C. Fluorescence intensities were continuously measured at 1-min intervals throughout the treatment period. The mitochondrial membrane potential (ΔΨm) was quantified as the ratio of red (aggregate) to green (monomer) fluorescence intensity [28]. All experiments were performed in three independent replicates.

### Measurement of intracellular and extracellular ATP level

An enhanced ATP assay kit (Beyotime; Shanghai, China) was applied to quantify intracellular and extracellular ATP levels, as described previously with modifications [29]. *C. albicans* SC5314 cells in RPMI-1640 medium at a concentration of 1 × 10^6^ CFU/mL were treated with PB at 2×MIC and 8×MIC at 35 °C for 4 h and then centrifuged to separate the supernatant from the cell pellet. Then, the supernatant was transferred into pre-chilled tubes for subsequent extracellular ATP detection. The cell pallet was resuspended with 200 μL of RPMI-1640 medium for the following OD_530_ detection and intracellular ATP assessment. The supernatant or the cell suspension was added into 100 μL of pre-chilled ATP detection solution and incubated on ice for 15 min. Luminescence was analyzed using a microplate reader (BioTek, VT, USA). ATP values were calculated from a standard curve for ATP increments and the results presented are the ATP values normalized to cell concentration. All experiments were performed in three independent replicates.

### Apoptosis determination

Hoechst 33,342 (Invitrogen) was applied to assess the apoptotic cells, as described previously with modifications [22]. *C. albicans* SC5314 cells in RPMI-1640 medium at a concentration of 1 × 10^6^ CFU/mL were treated with PB (64 μg/mL) or AMB at 4×MIC at 35 °C for 2 h. Following treatment, the suspension was centrifuged and the cell pellets were stained with 5 μg/mL Hoechst 33,342 at 35 °C for 30 min in the dark. Cells were visualized using a fluorescence microscope (Carl Zeiss). Apoptotic cells, characterized by chromatin condensation and nuclear fragmentation, displayed intense blue fluorescence, whereas viable cells exhibited dim and diffuse nuclear staining. One Step TUNEL Apoptosis Assay Kit (Beyotime; Shanghai, China) was also used to detect apoptotic cells according to the manufacturer’s instructions. *C. albicans* SC5314 cells were treated as described above and then the suspension was centrifuged and washed with PBS. Subsequently, the cells were permeabilized with an enhanced immunostaining permeabilization solution at room temperature for 10 min. After two washes with PBS, the cells were stained with TUNEL detection solution at 37 °C for 10 min. Apoptotic cells were identified by the presence of red fluorescence under a fluorescence microscope (Carl Zeiss). All experiments were performed in three independent replicates.

### Murine model of systemic candidiasis

The infection mouse model of systemic candidiasis was established as described previously with minor modifications [13], using *C. albicans* SC5314 to evaluate the antifungal efficacy of PB, both alone and in combination with AMB. Mice were obtained from the Animal Core Facility of Nanjing Medical University, and housed at the same place in a 12-h light – dark cycle with ambient temperature and humidity maintained at 68–74°F and 30–70%, respectively. Thirty 8-week-old specific-pathogen-free female C57BL/6 mice, acclimated for 1 week, were randomly assigned to five experimental groups. Body weights were monitored throughout the study period. Mice were anesthetized via intraperitoneal injection of 1% pentobarbital sodium (75 mg/kg) and subsequently infected via injection into the inner canthus venous plexus with 5.25 × 10^5^ CFU of *C. albicans* SC5314 suspended in 150 µL of PBS, with the exception of the uninfected control group. Two hours post-infection, treatment was initiated and administered for three consecutive days as follows: liposomal amphotericin B (L-AMB; 1.5 mg/kg) in 5% glucose solution was administered by tail vein injection once daily; PB (25 mg/kg) in a vehicle of 20% Solutol HS-15 and 0.5% Tween 80 (Vehicle) was administered by oral gavage twice daily; or a combination of both drugs. Mice in the uninfected and infected control groups received the corresponding vehicles according to the same schedule. Twenty-four hours after the final administration, mice were anesthetized via intraperitoneal injection of 1% pentobarbital sodium (75 mg/kg) euthanized by cervical dislocation. Kidneys and spleens were aseptically harvested, weighed, and homogenized in 1 mL of RPMI-1640 medium. Homogenates were serially diluted, plated on SDA agar, and incubated at 35°C for 24 h to determine viable fungal burdens. The remaining kidney tissue was fixed in formalin, embedded in paraffin, and sectioned for histopathological analysis using hematoxylin and eosin (H&E) and periodic acid – Schiff (PAS) staining. Tissue sections were examined by light microscopy (Carl Zeiss) to assess inflammatory infiltration, tissue architecture damage, and the presence of fungal hyphae.

The clinical isolate *C. albicans* C5 (VRC-resistant) was used to establish a murine model of systemic candidiasis to assess the potential synergistic effect of PB and VRC. Mice were infected as described above with 3.5 × 10^5^ CFU of *C. albicans* C5. Two hours post-infection, treatments were administered by oral gavage twice daily for 3 days: VRC (50 mg/kg), PB (25 mg/kg) in the same vehicle, or a combination of both drugs. Control groups received vehicle alone. All subsequent processing and analysis were performed as described above.

### Statistical analysis

GraphPad Prism 9 software was used for statistical analyses. Unpaired or one-sample t-test was used for comparisons between two groups or one group and a reference value, while one-way analysis of variance, followed by Tukey’s test or post hoc Dunnett’s test, for comparisons involving more than two groups. The values were presented as mean ± standard deviation. A *p*-value of less than 0.05 was considered as statistical significance. A *p*-value was calculated using the hypergeometric distribution method [30] to find the GO term with significantly enriched DEGs and identify the main biological functions associated with the DEGs.

## Results

### PB demonstrated antifungal activity against C. albicans and exhibited synergistic effects with conventional antifungals

To identify FDA-approved drugs with potential for repurposing in antifungal therapy, we screened a clinical compound library against a voriconazole-resistant *C. albicans* clinical isolate (strain C5). Screening at 25 µM in three independent experiments identified eight compounds that inhibited the growth of *C. albicans* C5: PB, clotrimazole, econazole nitrate, flucytosine, ciclopirox, piroctone olamine, terbinafine hydrochloride, and rapamycin (Table S1). Notably, PB was the only compound not previously recognized as an antifungal agent. PB exhibited broad-spectrum activity against *Candida* species, with MIC values ranging from 8 to 16 µg/mL against 17 *C. albicans* standard strains and clinical isolates (Table 1). It was also effective against *C. tropicalis*, *C. parapsilosis*, and *C. lusitaniae* (*Clavispora lusitaniae*) but showed relatively weak activity against *C. glabrata* (*Nakaseomyces glabratus*), *C. krusei* (*Pichia kudriavzevii*), and *C. auris* (*Candidozyma auris*) (Table 1).

**Figure 1.**
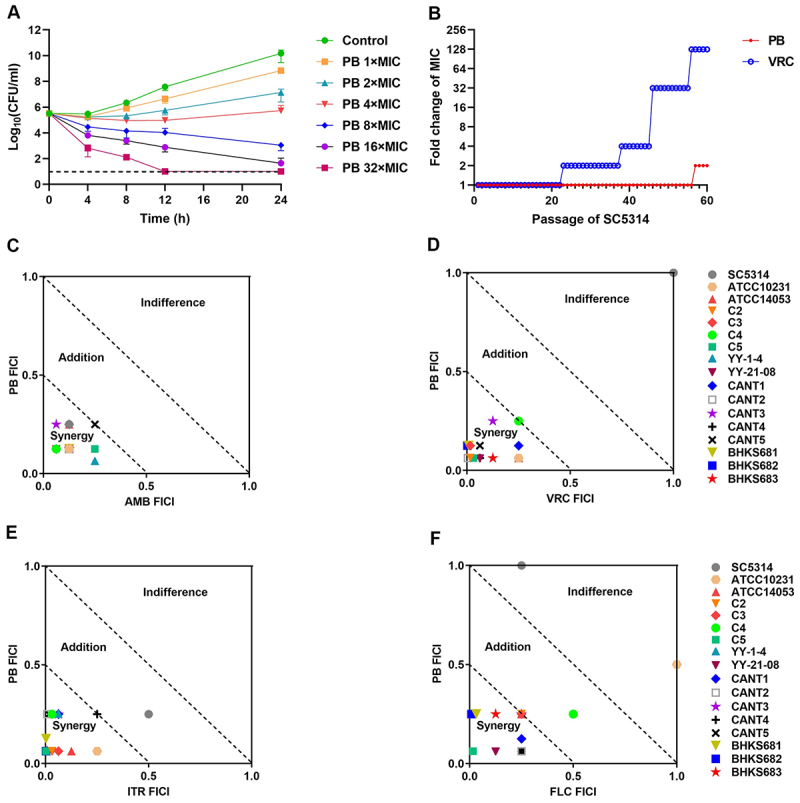
Anti-*C. albican*s property of PB. (A) kill kinetics of PB against *C. albicans* SC5314. If no colonies were observed, values were recorded using the limit of detection (10^1^ CFU/mL). Data are presented as mean ± SD from three independent replicates. (B) resistance development was conducted in the presence of sub-MIC concentration of PB, AMB, or VRC. (C)-(F) susceptibility of 17 *C. albicans* strains to PB in combination with AMB (C), VRC (D), ITR (E), and FLC (F) was determined by a checkerboard titration method.

Killing kinetics assessment revealed that PB exhibited both concentration- and time-dependent fungicidal activity. Treatment with PB at 2×, 4×, 8×, and 16×MIC led to 3.0-log10, 4.5-log_10_, 7.1-log_10_, and 8.5-log_10_ decreases in viable counts after 24 h, respectively (Figure 1A). Complete killing of *C. albicans* cells was observed when they were exposed to PB at 32×MIC within 12 h.

The potential for resistance development was assessed by serially passaging *C. albicans* SC5314 under sub-MIC PB pressure for 60 passages, using VRC as a control. While VRC exposure induced a 128-fold MIC increase by passage 56, PB exhibited only a twofold MIC increase after 56 passages (Figure 1B). This indicates a low propensity for resistance development against PB.

Checkerboard assays were then conducted to investigate the potential synergistic interaction between PB and conventional clinical antifungal agents. The combination of PB and AMB exhibited synergistic effects against all tested strains of *Candida* strains, with a fractional inhibitory concentration index (FICI) ranging from 0.188 to 0.5 (Figure 1C and Figure S1). This synergy was further confirmed by kinetic killing assays of PB in combination with AMB against *C. albicans* SC5314 (Figure S2). Neither PB at 64 μg/mL nor AMB at 0.5 μg/mL alone exhibited fungicidal activity. However, their combination resulted in a significant fungicidal effect, leading to an approximately 4-log_1__0_ CFU/mL reduction after 12 h compared to either monotherapy. Additionally, PB also demonstrated synergistic effects with azoles, including VRC, ITR, and FLC, against most of the tested *C. albicans* strains (94.1%, 94.1%, and 88.2% respectively) (Figure 1D-1F).

### PB inhibited the yeast-to-hypha transition and biofilm formation of C. albicans

**Figure 2.**
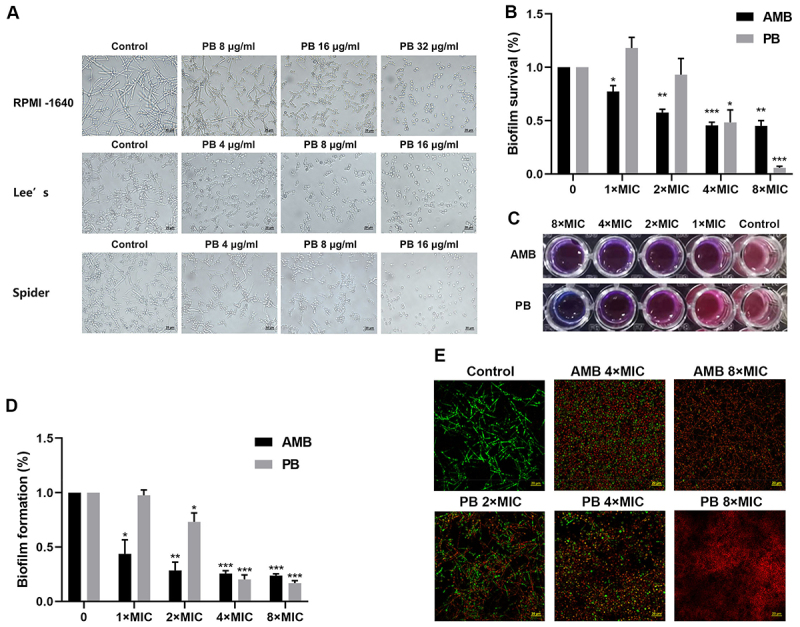
PB inhibited the yeast-to-hypha transition and biofilm formation in *C. albicans*. (A) hyphal development of *C. albicans* SC5314 under inducing conditions (RPMI-1640, Lee’s, or Spider medium) in the presence of various PB concentrations. (B) inhibition of biofilm formation quantified by crystal violet staining. (C, D) metabolic activity of cells within pre-formed mature biofilms after PB treatment, assessed by Alamar Blue assay; (C) provides quantitative fluorescence measurements, while (D) shows qualitative results. (E) CLSM images of mature biofilms stained with SYTO-9 (live cells, green) and PI (dead cells, red) following PB treatment. Data are from three independent replicates and presented as mean ± SD. Statistical significance versus the untreated control was determined by a one-sample t-test (*p < 0.05, **p < 0.01, ***p < 0.001). Scale bar: 20 μm.

The yeast-to-hypha transition in *C. albicans* is a critical virulence determinant that facilitates host cell adhesion and invasion [31]. To assess the impact of PB on hyphal morphogenesis, we cultured *C. albicans* SC5314 in hypha-inducing media (RPMI-1640, Lee’s, and Spider). While robust hyphal development was observed in untreated controls, PB treatment markedly suppressed hyphal growth. At 16 µg/mL and 32 µg/mL, PB nearly abolished hyphal formation across all tested media (Figure 2A), demonstrating its potent inhibitory effect.

Biofilm formation by *C. albicans* represents a major clinical challenge, driving persistent infections and underscoring the need for novel antifungal strategies [32]. Crystal violet staining revealed that PB significantly inhibited biofilm biomass accumulation at concentrations exceeding 2×MIC (Figure 2B). Alamar Blue was employed to evaluate the effect of PB on the cell viability within mature biofilms. The results showed that PB significantly reduced cell viability within mature biofilms in a concentration- dependent manner, achieving almost complete inhibition at 8×MIC (Figure 2C and 2D). This reduction in viability was corroborated by SYTO-9/propidium iodide (PI) staining. When observed under a confocal laser scanning microscope (CLSM), viable cells of *C. albicans* SC5314 in the control group exhibited green fluorescence (SYTO-9 staining), while PB at 8×MIC killed almost all the *C. albicans* cells within the biofilms, as evidenced by red fluorescence (PI staining) (Figure 2E). As a positive control, AMB also demonstrated killing activity against biofilm cells. Collectively, these results highlight PB’s potential as an antibiofilm agent against *C. albicans*.

### PB induced mitochondrial dysfunction, apoptosis and iron homeostasis perturbation in C. albicans

Previous studies have reported that PB can inhibit Ca^2+^ influx through the L-type calcium channel (LTCC) of smooth muscle cells [33] and bind to the human voltage-gated calcium channel Ca_v_1.2, a predominant isoform of LTCC [34]. To determine if a similar calcium-mediated mechanism contributes to its antifungal activity, we first assessed the effect of PB on intracellular calcium levels ([Ca^2 +^]_i_) in *C. albicans*. Our results showed that PB treatment did not alter [Ca^2 +^]_i_ compared to the control (Figure S3). Furthermore, genomic analysis revealed no homologues of the Ca_v_1.2 channel in *C. albicans*. These findings demonstrate that PB’s antifungal action is not mediated through the inhibition of calcium channels.

**Figure 3.**
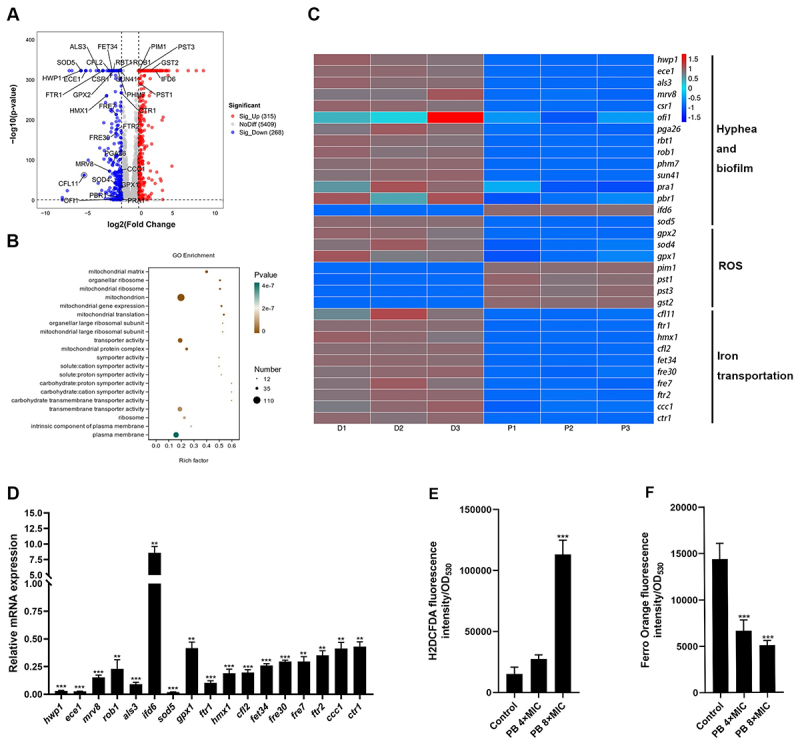
Mechanistic insights into the antifungal activity of PB against *C. albicans*. (A) volcano plot and (B) GO enrichment analysis of DEGs following PB treatment compared to the control. DEGs were defined by a |log2Fold change| >1. (C) heatmap depicting the expression patterns of selected DEGs related to hyphal development, biofilm formation, ROS response, and iron transport. (D) validation of a subset of these DEGs by RT-qPCR. (E) intracellular ROS accumulation measured by H_2_DCFDA staining. (F) intracellular ferrous iron Fe^2 +^ levels assessed using Ferro Orange staining. Data are presented as mean ± SD from three independent replicates. Statistical significance was determined using a one-sample or unpaired t-test (*p < 0.05, **p < 0.01, ***p < 0.001).

We then performed transcriptomic analysis on *C. albicans* SC5314 exposed to PB or a DMSO control. This analysis identified 583 differentially expressed genes (DEGs) in the PB-treated group compared to the control, comprising 315 upregulated and 268 downregulated genes (Figure 3A and Figure S4A), and hierarchical clustering was performed (Figure S4B). Gene Ontology (GO) enrichment analysis revealed significant enrichment of biological processes associated with mitochondrial processes, including mitochondrial matrix, gene expression, translation, and protein complex formation (Figure 3B). The upregulation of these genes may represent a compensatory response to mitochondrial dysfunction. Furthermore, genes associated with hyphal and biofilm formation (*hwp1*, *ece1*, *als3*, *mrv8*, *csr1*, *ofi1*, *pga26*, *rbt1*, *rob1*, *phm7*, *sun41*, *pra1*, *pbr1* and *sod5*) were predominantly downregulated (Figure 3C), consistent with the observed inhibition of hyphae and biofilm formation by PB. The exception was *ifd6*, a negative regulator of biofilm formation [35], which was upregulated.

Given that antifungals such as AMB and azoles exert their effects through reactive oxygen species (ROS) generation, we examined oxidative stress pathways. Transcriptomic data indicated that PB altered the expression of oxidative stress-related genes, downregulating *gpx2*, *sod4*, and *gpx1* while upregulating *pim1*, *pst1*, *pst3* and *gst2*. Some of these DEGs were validated by quantitative real-time polymerase chain reaction (RT-qPCR) (Figure 3D). Consequently, we assessed intracellular ROS levels using a 2ʹ,7ʹ-dichlorofluorescin-diacetate (H_2_DCFDA) fluorometric assay, which confirmed that PB induces significant ROS accumulation (Figure 3E), suggesting it as a potential antimicrobial mechanism.

**Figure 4.**
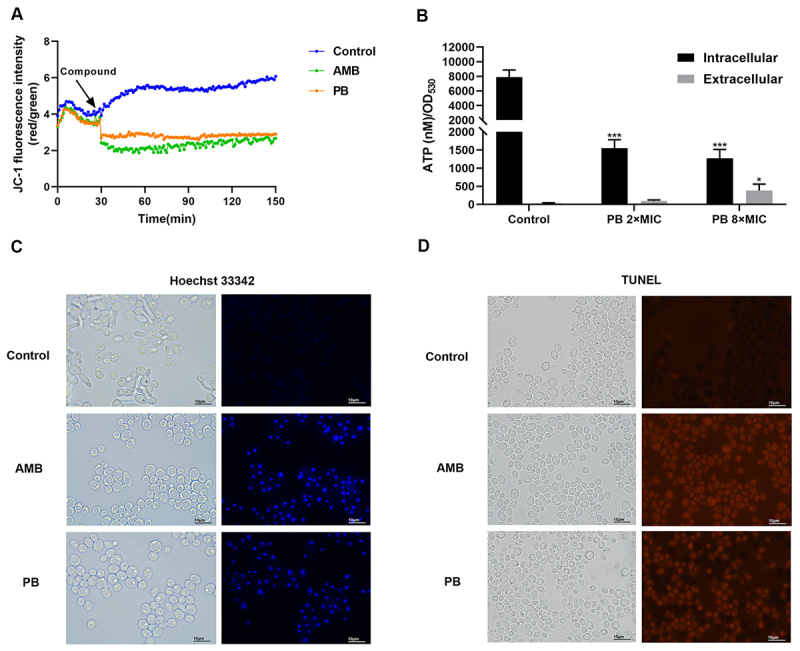
PB disrupts mitochondrial function and induces apoptosis in *C. albicans*. (A) mitochondrial membrane potential measured by JC-1 staining and expressed as the red/green fluorescence intensity ratio. (B) intracellular and extracellular ATP levels quantified using an enhanced ATP assay kit. Data are presented as mean ± SD from three independent replicates. Statistical significance was determined by an unpaired t-test comparing treated groups to the control (*p < 0.05, ***p < 0.001). (C, D) apoptosis assessment via Hoechst 33,342 staining (C), indicating nuclear condensation, and (D) TUNEL assay, indicating DNA fragmentation. Scale bar: 10 μm.

Since ROS can disrupt mitochondrial membrane potential (ΔΨm) [36], we utilized the JC-1 dye to evaluate ΔΨm. PB treatment, like AMB, caused a dissipation of ΔΨm, indicated by a shift from red to green fluorescence, confirming mitochondrial dysfunction (Figure 4A). As mitochondria are the primary site of ATP synthesis [37], we next measured intracellular ATP levels, which were significantly reduced following PB exposure (Figure 4B). This decrease aligns with the observed mitochondrial damage. A concurrent increase in extracellular ATP suggests an additional compromise to cell membrane integrity.

The consumption of mitochondrial membrane potential is a crucial cellular event during early apoptosis [38]. To investigate this, we used Hoechst 33,342 staining, which revealed chromatin condensation (a key apoptotic feature) in PB-treated cells, an effect comparable to that of AMB (Figure 4C). Apoptosis was further confirmed by TUNEL assay, which detected DNA fragmentation (red fluorescence) in PB- and AMB-treated cells (Figure 4D).

GO analysis also indicated enrichment in transporter activity, specifically implicating genes involved in iron ion transport (*ftr1, ftr2, ccc1, ctr1*) and broader iron homeostasis (*als3*, *gfl11*, *hmx1*, *cfl2*, *fet34*, *fre30*, and *fre7*) (Figure 3C). Some of these genes regulating iron homeostasis were validated by RT-qPCR (Figure 3D). To functionally assess iron homeostasis, we employed the FerroOrange probe, which detects intracellular free Fe^2 +^. PB treatment resulted in a concentration-dependent depletion of free Fe^2 +^ (Figure 3F), corroborating the gene expression data related to impaired iron uptake.

To identify potential antimicrobial targets of PB, we performed batch molecular docking against the *C. albicans* SC5314 proteome. The top-scoring candidates included an uncharacterized protein (CAALFM_C504940WA), a cyclin-dependent serine/threonine protein (CAALFM_C302260CA), a transcription factor Sef1 (CAALFM_CR02190CA), and another uncharacterized protein (CAALFM_CR06640CA) (Figure S5A). While the uncharacterized proteins and the kinase represent interesting subjects for future investigation, we focused on Sef1 because it is a known master regulator of iron uptake in *C. albicans* [39], and our transcriptomic and functional data (Figure 3C, 3D, 3F) strongly implicated iron homeostasis disruption as a mechanism of PB action. Docking analysis predicted that PB binds effectively to Sef1 via hydrophobic interactions with Pro334, Lys332, Val339, and Thr328, and a hydrogen bond with Val392 (Figure S5B). This strong binding interaction may impede the nuclear translocation of Sef1 and its subsequent activation of transcriptional programs, ultimately inhibiting iron acquisition and contributing to the antifungal efficacy of PB.

### PB and AMB synergistically induced ROS accumulation and caused damage to the cell membrane

**Figure 5.**
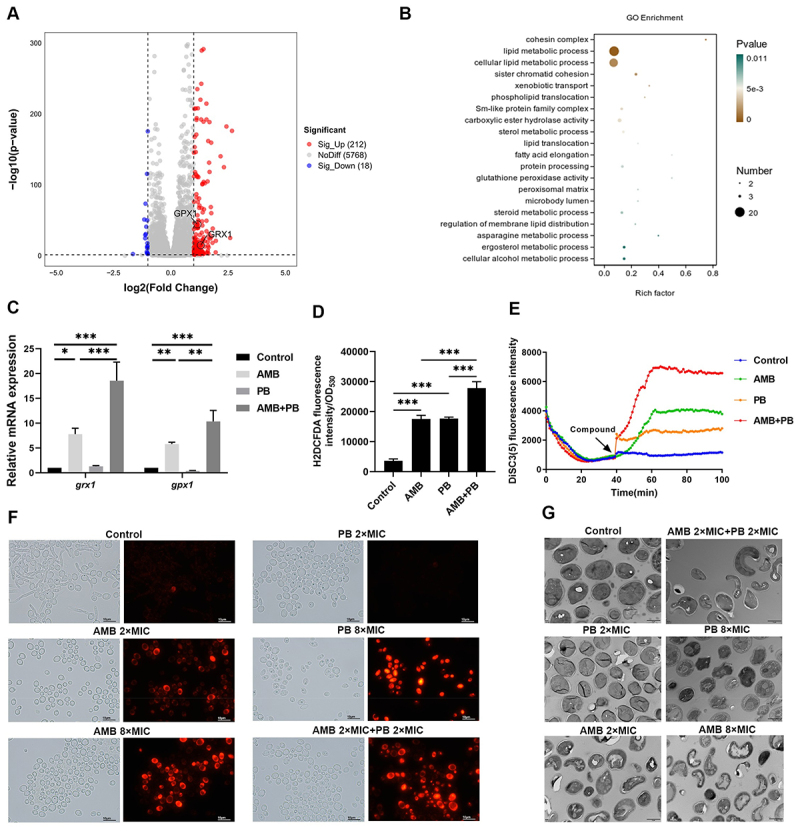
Mechanism of synergistic action between PB and AMB against *C. albicans*. (A and B) volcano plot (A) and functional enrichment diagram (B) displaying the distribution and GO enrichment analysis of DEGs in the combination treatment group (PB + AMB) relative to the AMB-treated group. DEGs were identified using a threshold of |log_2_ Fold change| >1. (C) RT-qPCR validation of the expression levels of two ROS-related genes, *grx1* and *gpx1*. (D) intracellular ROS generation measured by H_2_DCFDA staining. Data are presented as mean ± SD from three independent biological replicates and were analyzed by one-way ANOVA with Tukey’s post-hoc test. *, *p* < 0.05; **, *p* < 0.01; ***, *p* < 0.001. (E) alterations in mitochondrial membrane potential monitored using the DiSC_3_(5) fluorescent probe. (F) assessment of membrane permeabilization via PI staining and fluorescence microscopy. (G) ultrastructural morphological changes observed by TEM. Scale bars: 10 µm (F) and 2 µm (G).

To elucidate the mechanisms underlying this synergy between PB and AMB, we performed transcriptomic analysis on *C. albicans* SC5314 following exposure to PB, AMB, or their combination. The profile of DEGs were shown in Figure S4. Compared to the control, the AMB monotherapy group exhibited 3660 DEGs (1798 upregulated, 1862 downregulated), while the combination therapy group exhibited 3575 DEGs (1712 upregulated, 1863 downregulated). Significantly, when compared with the AMB- treated group, a total of 240 DEGs were detected in *C. albicans* in the dual therapy group, including 212 upregulated and 18 downregulated genes (Figure 5A and Figure S4A). GO enrichment analysis of these DEGs indicated significant enrichment of biological processes related to lipid and steroid metabolism in the combination group. These processes included lipid metabolic process, lipid translocation, regulation of membrane lipid distribution, phospholipid translocation, fatty acid elongation, and sterol, steroid, and ergosterol metabolic processes (Figure 5B). Additionally, GO terms associated with redox reactions, such as glutathione peroxidase activity and peroxisomal matrix, were enriched. Specifically, the expression of two key oxidative stress-related genes—*gpx1* (encoding glutathione peroxidase-1, an antioxidant enzyme induced under oxidative stress) [40,41] and *grx1* (encoding a cytoplasmic thiol-disulfide oxidoreductase that maintains redox homeostasis) [42]—was upregulated by AMB and further enhanced by the combination therapy. These two genes, indicated in the volcano plot, were validated by RT-qPCR (Figure 5C). These findings suggest that the antifungal synergy may involve intracellular ROS accumulation and disruption of cell membrane homeostasis. Consistent with the transcriptomic data, H_2_DCFDA fluorescence assays confirmed that both PB and AMB monotherapies significantly enhanced ROS levels, an effect that was synergistically augmented by the dual therapy (Figure 5D).

As ROS can mediate cellular damage by disrupting the cell membrane [43], we assessed membrane potential using the lipophilic dye DiSC_3_(5). Both drugs alone induced depolarization, and this effect was significantly enhanced by their combination (Figure 5E). We further investigated membrane integrity using propidium iodide (PI) staining, which fluoresces upon binding nucleic acids in cells with compromised membranes. While a concentration of 8×MIC for either drug alone was required to cause a clear increase in PI uptake, the combination therapy at just 2×MIC significantly enhanced membrane permeabilization (Figure 5F), demonstrating a synergistic disruptive effect on the cell membrane. Untreated cells displayed no red fluorescence, confirming membrane integrity.

Given the induction of ROS and membrane damage, we utilized transmission electron microscopy (TEM) to examine morphological and ultrastructural alterations. Untreated cells exhibited a round morphology with intact cell walls, dense cytoplasm, and distinct organelles (Figure 5G). Cells treated with PB at 2×MIC showed no obvious changes, but at 8×MIC, they displayed deformed cell walls, dented membranes, expanded vacuoles, and cytoplasmic cavitation. AMB treatment also caused malformations of the cell wall and membrane, with 8×MIC leading to severe cytoplasmic disintegration and organelle disruption. Notably, fungal cells treated with the dual therapy at 2×MIC exhibited a distorted shape, a rugged surface, swollen vacuoles, and barely visible organelles (Figure 5G), indicating potent synergistic candidacidal activity.

### PB performed antifungal effect and synergized with AMB and VRC in mouse models of systemic candidiasis

**Figure 6.**
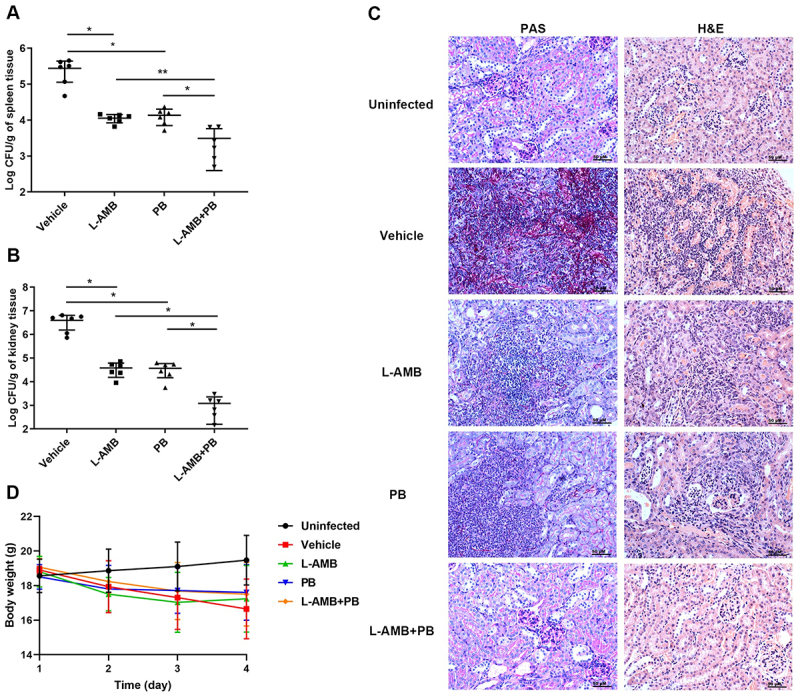
*In vivo* efficacy of PB alone and in combination with AMB in a murine model of systemic *C. albicans* SC5314 infection. (A, B) quantification of fungal burden in the spleen (A) and kidney (B) of the infected mice treated with vehicle, L-AMB, PB, or L-AMB + PB. Error bars represent the standard deviations derived from six mice per group. Statistical differences between two groups were analyzed by a one-way ANOVA with post hoc Dunnett’s test for multiple comparisons (*p < 0.05, **p < 0.01). (C) representative micrographs of kidney sections stained with PAS and H&E following the indicated treatments. Scale bar: 50 µm. (D) changes in body weight of mice over the course of treatment.

To evaluate the therapeutic effect of PB alone and in combination with AMB *in vivo*, a systemic candidiasis model was established by intravenous infection of mice with *C. albicans* SC5314. Given the established reduction in nephrotoxicity associated with lipid formulations of AMB, amphotericin B liposomes (L-AMB) were used in this study. As shown in Figure 6A and 6B, both PB and L-AMB monotherapy exhibited significant antifungal activity *in vivo*. PB monotherapy reduced fungal burden in the spleen and kidneys by approximately 1.25-log_10_ and 2.02-log_10_ CFU, respectively. Similarly, L-AMB monotherapy yielded reductions of 1.29-log_10_ and 1.98-log_10_ CFU in these organs. In contrast, combination therapy induced a markedly greater reduction in fungal load, achieving 2.02-log_10_ and 3.58-log_10_ CFU decreases in the spleen and kidneys, respectively. These decreases were statistically significant compared to the monotherapy groups, confirming a synergistic interaction between PB and L-AMB *in vivo*.

These findings were corroborated by histopathological analysis. Periodic acid-Schiff (PAS) staining of kidney sections revealed extensive hyphal invasion (stained red) in the vehicle controls (untreated) (Figure 6C). While PB or AMB monotherapy inhibited hyphal formation, hyphae were nearly absent in kidneys from the combination therapy group. Hematoxylin and eosin (H&E) staining further supported the fungal burden results (Figure 6C). Both monotherapies attenuated inflammatory cell infiltration and tissue damage induced by *C. albicans*. This protective effect was enhanced by combination therapy, which resulted in relatively preserved tissue architecture and minimal inflammation. In addition, all infected mice exhibited minimal weight loss with no significant differences between the vehicle control and each treated groups, indicating low drug toxicity (Figure 6D).

**Figure 7.**
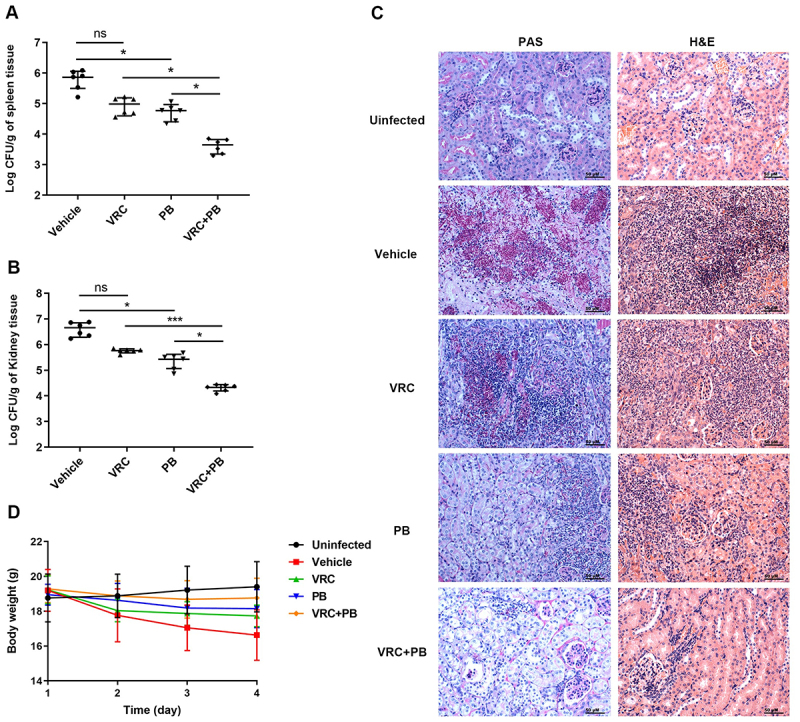
*In vivo* efficacy of PB alone and in combination with VRC in a murine model of systemic *C. albicans* C5 infection. (A, B) quantification of fungal burden in the spleen (A) and kidney (B) of the infected mice treated with vehicle, VRC, PB, or VRC + PB. Error bars represent the standard deviations derived from six mice per group. Statistical differences between the two groups were analyzed by a one-way ANOVA with post hoc Dunnett’s test for multiple comparisons (*p < 0.05, **p < 0.001, ns, no significant difference). (C) representative micrographs of kidney sections stained with PAS and H&E following the indicated treatments. Scale bar: 50 µm. (D) changes in body weight of mice over the course of treatment.

The efficacy of PB against azole-resistant strains was evaluated using a systemic candidiasis model established with the VRC-resistant clinical isolate *C. albicans* C5. PB monotherapy demonstrated significant antifungal activity against this VRC-resistant strain, reducing splenic and renal fungal burdens by approximately 1.07-log_10_ and 1.24-log_10_ CFU, respectively (Figure 7A and 7B). Critically, PB restored susceptibility to VRC, as combination therapy achieved substantial reductions of 2.33-log_10_ CFU in the spleen and 2.27-log_10_ CFU in the kidney, demonstrating more potent antifungal activity than either monotherapy. Histopathological assessment of PAS- and H&E-stained kidney tissues confirmed these results (Figure 7C), showing reduced fungal invasion and tissue pathology consistent with the observed decreases in fungal burden. In agreement with previous results, all infected mice showed minimal weight changes, indicating low toxicity (Figure 7D).

## Discussion

Previous studies have reported that various existing drugs, including statins, phenothiazines, and antidepressants, exhibit utility in the treatment of *Candida* infections [10]. In this study, we identified PB, a clinically approved antispasmodic drug, as a novel antifungal agent through screening a library of FDA-approved compounds. PB demonstrated broad-spectrum antifungal activity, particularly against *C. albicans*. Beyond direct growth inhibition, PB significantly suppressed key *C. albicans* virulence factors, including hyphal formation and biofilm development, while exhibiting a low propensity to induce resistance. Furthermore, PB demonstrated synergistic interactions with AMB and azole antifungals against *C. albicans* both *in vitro* and *in vivo*. Mechanistic investigations revealed that PB potentiates AMB activity by augmenting ROS accumulation and increasing membrane permeability. This combination strategy may offer advantages over another antispasmodic drug, otilonium bromide, which has also been reported to exhibit antifungal activity against *C. albicans* [44]. In addition, PB disrupts mitochondrial function, induces apoptosis, and critically inhibits cellular iron uptake. Interestingly, PB has also been demonstrated to possess antibacterial activity against *Staphylococcus aureus* and *Staphylococcus epidermidis* [45,46]. Collectively, these findings highlight the therapeutic potential of PB for antifungal applications.

Synergistic combinations of current antibiotics with bioactive compounds represent a promising strategy for enhancing antimicrobial treatment efficacy [47,48]. Our findings strongly support the therapeutic potential of PB combined with azoles or AMB, warranting clinical evaluation. Notably, in two mouse models of systemic *C. albican*s infections, PB co-administration significantly enhanced the efficacy of VOR and AMB. As well, PB was well tolerated in mice, indicating a favorable safety profile. Future investigations will focus on developing optimized formulations for combination delivery and elucidating the pharmacokinetic properties of PB. These studies aim to facilitate effective administration in combination regimens and characterize the compound’s distribution, metabolism, and elimination profiles.

The pathogenic potential of *C. albicans* is intrinsically linked to its dimorphic switch and biofilm formation [49]. Hyphal growth facilitates tissue invasion and adhesion, representing a critical transition from commensalism to pathogenicity [5]. Biofilm formation confers substantial resistance to antifungals, mediated by factors such as increased cell density, quorum sensing, extracellular matrix production, persister cells, efflux pump upregulation, and enhanced stress responses [50]. Our data demonstrate that PB not only inhibits hyphal initiation and nascent biofilm formation but also disrupts mature biofilms, indicating its capacity to mitigate *C. albicans* pathogenesis. Although transcriptomic and RT-qPCR analyses confirmed PB-mediated downregulation of hyphal and biofilm-associated gene expression, the detailed molecular mechanisms remain incompletely characterized.

Transcriptomic analysis also revealed that PB significantly downregulates the expression of genes involved in iron acquisition. Iron is indispensable for *C. albicans* growth, metabolism, and virulence [51,52], serving as a cofactor in critical processes such as respiration and DNA synthesis. Cellular iron homeostasis involves storage in vacuoles and mitochondria, regulated by the Mrs4-Ccc1-Smf3 pathway [51], where Ccc1 imports cytoplasmic iron into vacuoles [53]. In this study, the expression of *ccc1* was downregulated following PB treatment, indicating reduced iron transportation into vacuoles for storage. In addition, *C. albicans* acquires iron through multiple pathways: a reductive iron assimilation (RIA) pathway mediated by cell surface ferric reductases (e.g. Fre7, Fre30, Cfl2, Cfl11) converting Fe^3 +^ to Fe^2 +^ [54,55]; a haeme-iron uptake system involving Rbt5 and the heme oxygenase Hmx1 [56]; a siderophore uptake system involving multicopper oxidases (e.g. Fet34) re-oxidizing Fe^2 +^ requiring the copper transporter Ctr1 [57,58]; and iron permeases (Ftr1, iron-deprivation induced; Ftr2, iron-replete induced) transporting Fe^3 +^ [59]. Our study showed that PB suppresses the expression of all these genes, which correlated with decreased intracellular Fe^2 +^ levels. Furthermore, PB appeared to bind to the transcription factor Sef1, potentially impairing its nuclear translocation and activation of iron uptake genes-an interaction warranting further genetic and biochemical validation. Iron limitation is known to inhibit hyphal growth and biofilm formation [60,61], induce ROS accumulation and apoptosis [62,63], cause mitochondrial dysfunction [64], and enhance membrane fluidity and drug susceptibility [59,65]. These established consequences align precisely with our observations of PB’s effects.

A critical consideration for drug repurposing is whether the effective concentration in preclinical models translates to achievable and safe exposures in humans. In our murine model of systemic candidiasis, PB was administered at 25 mg/kg twice daily. Based on body surface area conversion, this dose corresponds to a human equivalent of approximately 4 mg/kg/day, or roughly 240 mg/day for a 60 kg adult. This falls within the established clinical dose range of PB (150–300 mg/day) approved for functional gastrointestinal disorders. However, PB is known to have low oral bioavailability in humans, raising the question of how systemic antifungal efficacy is achieved. We propose two non-mutually exclusive mechanisms to reconcile this apparent pharmacokinetic paradox. First, although systemic absorption is limited, the absorbed fraction may preferentially accumulate in reticuloendothelial organs such as the kidneys, spleen, and liver in mice, potentially achieving local tissue concentrations sufficient to exert direct antifungal effects. Second, PB may act indirectly through host-directed immunomodulation, for instance by modulating the gut microbiota or gut-associated lymphoid tissue (GALT), thereby enhancing systemic host defense against *C. albicans*. The human safety profile of PB, together with its potent *in vivo* activity in murine models, provides a strong rationale for further evaluation of its antifungal potential, particularly in combination therapy. However, we acknowledge that body weight monitoring and histopathological examination of kidney organ alone constitute an incomplete safety assessment, particularly for a drug being considered for systemic antifungal use. Comprehensive toxicological evaluation, including histopathological examination of major organs (e.g. liver, kidney, heart), serum biochemical markers of hepatic and renal function, and hematological parameters, is necessary to fully define the safety window of PB. Such studies are planned as part of our ongoing efforts to advance PB toward potential clinical application as an antifungal agent.

In conclusion, our findings suggested the potential of PB for antifungal applications, either alone or in combination with conventional drugs. Additionally, targeting iron homeostasis and ROS accumulation might be a promising strategy for the treatment of *C. albicans* infection.

## Supplementary Material

supplementary files clean.docx

Ethics approval.pdf

Author Checklist.pdf

## Data Availability

The Supplementary materials can be available in the online repository Figshare at https://doi.org/10.6084/m9.figshare.32083554 (DOI: 10.6084/m9.figshare.32083554) [66]. The raw RNA-seq data presented in this research have been deposited in the National Center for Biotechnology Information BioProject database (https://www.ncbi.nlm.nih.gov/sra) and are available in the Sequence Read Archive (SRA) (PRJNA1212472). The data that support the findings of this study are available in the online repository Figshare at https://doi.org/10.6084/m9.figshare.31043158 (DOI: 10.6084/m9.figshare.31043158) [67].
