# Supplementary material for: Repurposing the antispasmodic drug pinaverium bromide as a novel antifungal agent and synergist against Candida albicans
Source: Virulence. 2026 Jun 1;17(1):2682573. doi: 10.1080/21505594.2026.2682573 (PMC13232878; doi:10.1080/21505594.2026.2682573)
Supplement: supplementary files clean.docx [file KVIR_A_2682573_SM8097.docx]

Supplementary Materials for

**Repurposing the antispasmodic drug pinaverium bromide as a novel antifungal agent and synergist against *Candida albicans***

Jing Yao^a,b,c,#^, Zhiyu Yang^c,#^, Xudong Hang^a^, Tianyu Chen^d^, Binglei Li^c^, Ting Shi^c^, Keao Quan^c^, Jingchen Xu^c^, Liping Zeng^c^, Ganzhu Feng^b*^, Hongkai Bi^a,c*^

^a^NHC Key Laboratory of Tropical Disease Control, School of Life Sciences and Medical Technology, Hainan Medical University, Haikou, Hainan, China

^b^Department of Respiratory and Critical Care Medicine, The Second Affiliated Hospital of Nanjing Medical University, Nanjing, Jiangsu, China

^c^Department of Pathogen Biology, Jiangsu Key Laboratory of Pathogen Biology, Nanjing Medical University, Nanjing, Jiangsu, China

^d^Department of Respiratory and Critical Care Medicine, The Affiliated Taizhou People’s Hospital of Nanjing Medical University, Taizhou, Jiangsu, China

^#^ These authors contribute equally to this work.

^*^Corresponding author

E-mail: hkbi@muhn.edu.cn (HB); fgz62691@163.com (GF).

**Table S1. Library Detailed Information**

**Table S2.** Primer sequences used in this study.

| **Primer** | **Sequence** |
| --- | --- |
| ACT1-F | AAGAATTGATTTGGCTGGTAGAGA |
| ACT1-R | TGGCAGAAGATTGAGAAGAAGTTT |
| HWP1-F | TGTCTACACTACATTCTGTC |
| HWP1-R | AGGAATAGATGGTTGTGAAC |
| ECE1-F | ATCGAAAATGCCAAGAGAG |
| ECE1-R | AGCATTTTCAATACCGACAG |
| MRV8-F | ACACCGACGATTGGACTGAA |
| MRV8-R | AGCAGGAATGAGACCCCAAC |
| ROB1-F | ACCAGTTTTCCCAACTTCAGGA |
| ROB1-R | AGTTGTGGCTCCAAATGCCA |
| ALS3-F | ATTCGATCCTAACCGCGACA |
| ALS3-R | TTGGTGCAGTTTTGGTCAGGT |
| IFD6-F | GTGGTGGTGTCTTGTGTCGT |
| IFD6-R | TCACGAGGTTCACCCAATCC |
| SOD5-F | ACTTTGCTTGACGAGGGACA |
| SOD5-R | CAGCGCCATTACCTTGAGGA |
| FTR1-F | GTGGTAGTTCCGACCCTCAA |
| FTR1-R | GCACCGATGAAAGCACAACC |
| HMX1-F | TGACGTTAAATGTGTGGCTGA |
| HMX1-R | ACCAAGGCAGCATAGTAGCC |
| CFL2-F | TTGAGGCTTCTGGCCGTAAA |
| CFL2-R | TGGATGCACACACAGTACCA |
| FET34-F | GGTAGCAGCAGAGACGCATA |
| FET34-R | CTCAAGGTGGGTAAAGGCCA |
| FRE30-F | TCTGTCCCTCTCAAGGTCCC |
| FRE30-R | ACGACGCACCATAGTAGACC |
| FRE7-F | AAGAAGGGTGCAAACGGAGA |
| FRE7-R | TCCTTTTGCGTAGCCTGTGT |
| FTR2-F | CTCAGCAAACGGTCCAGAGT |
| FTR2-R | CTCAGCAAACGGTCCAGAGT |
| CCC1-F | CGCTTGGGGATTGGTGTTCT |
| CCC1-R | TCGTTTTAAGTGATGTTGTTTCGGA |
| CTR1-F | CACAAAAGCTCGTGGAACCG |
| CTR1-R | AGCAGCCAACATCAAGGAGT |
| GRX1-F | GCTGATTCTTGCCTGGGGAT |
| GRX1-R | TGGTTTGGTCACAAAATGGACA |
| GPX1-F | GTTGCCTCCAGATGTGGGTT |
| GPX1-R | TTAGTGCCTGGTTCTTGCCA |


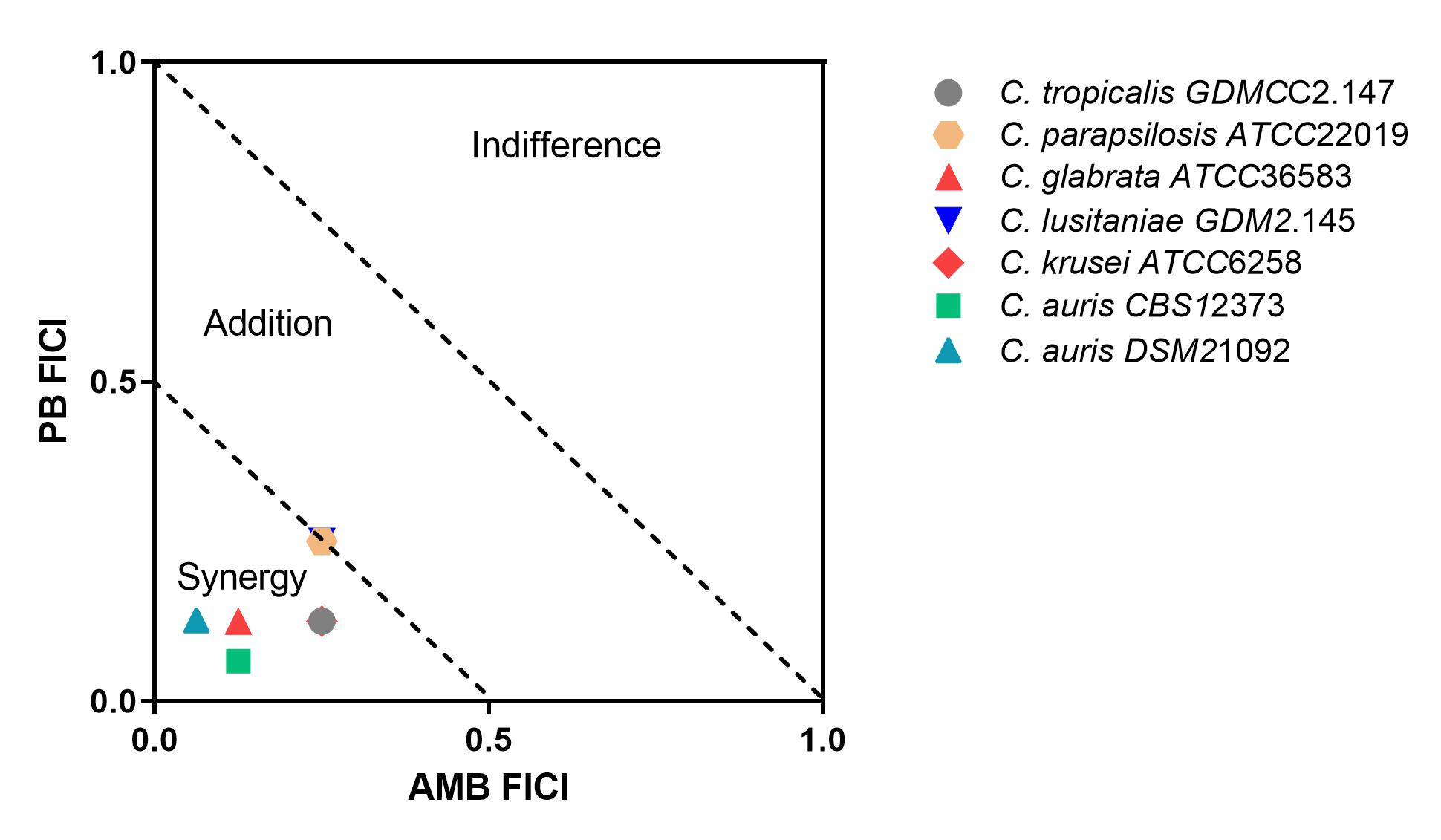


**Figure S1.** PB acts synergistically with AMB against fungal strains. Checkerboard titration method was applied and the FICI was calculated to assess the interaction between AMB and PB.

**Figure S2**. Time-kill curve analyses were performed by culturing *C. albicans* SC5314 in RPMI 1640 medium in the presence of PB (64 μg/mL), AMB (0.5 μg/mL), or the PB (64 μg/mL) + AMB (0.5 μg/mL) combinations. If no colonies were present, calculations were made using the limit of detection (10^1^ CFU/mL). Data represent medians ± standard deviations of the results from three independent experiments.


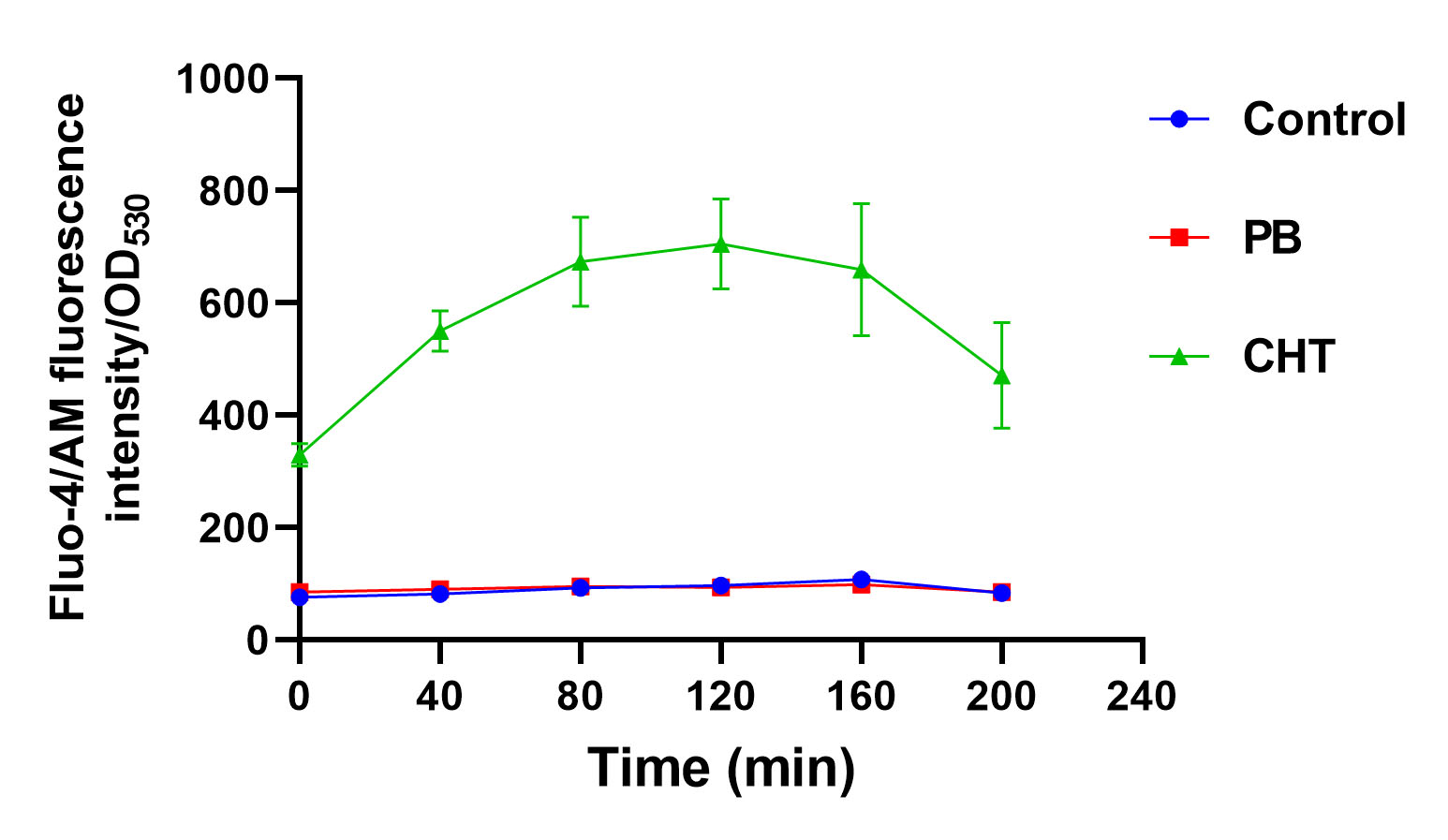


**Figure S3.** Effect of PB on [Ca^2+^]_i_. Yeast cells were stained with the fluorescent probe Fluo-4 AM and then treated with drugs. Mean fluorescence intensity was measured according to the Methods. Results represent the average of three independent experiments ± standard deviation. CHT: chelerythrine. As a positive control, CHT increased [Ca^2+^]_i_ in yeast cells [1].


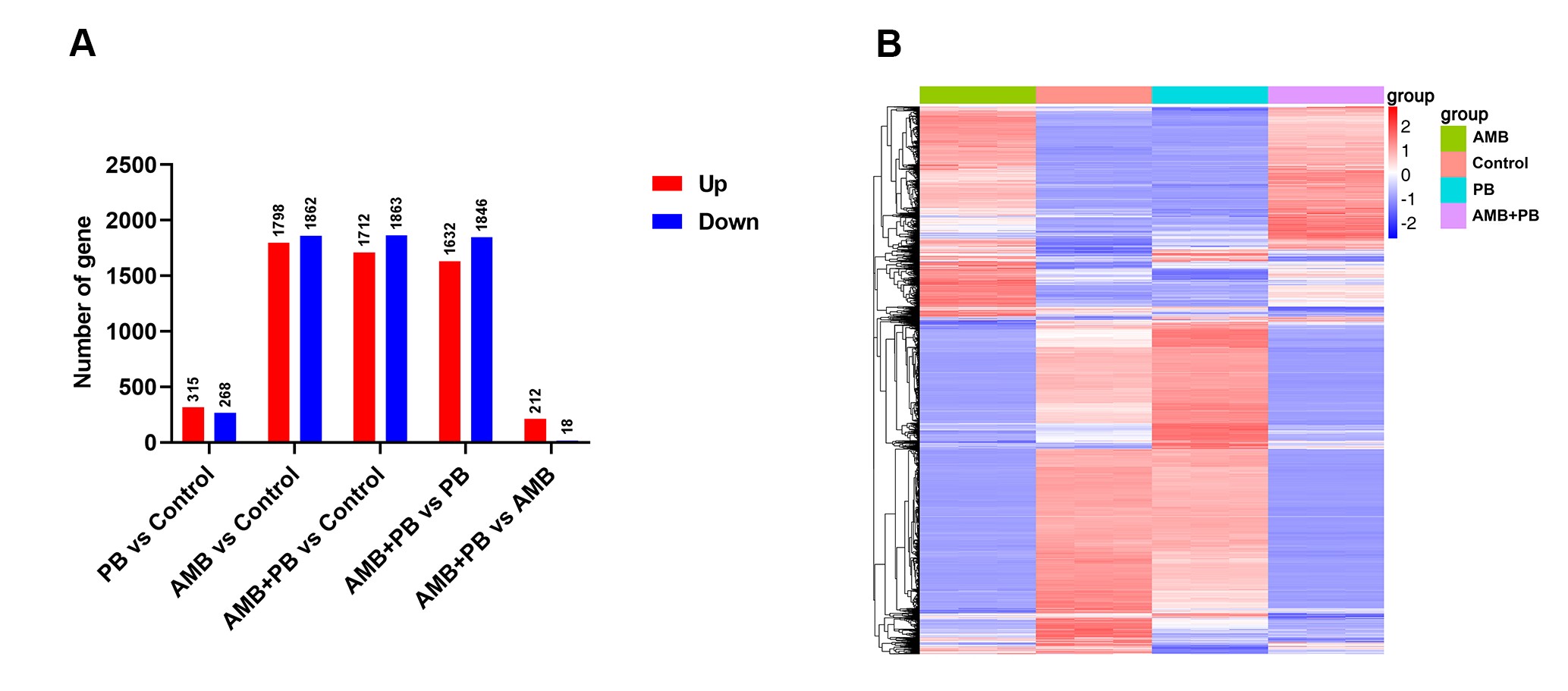


**Figure S4.** Transcriptomic analysis of *C. albicans* SC5314 treated with PB, AMB or the combination. The numbers (A) and the heat map of hierarchical clustering (B) of differentially expressed genes after drug treatment were presented.


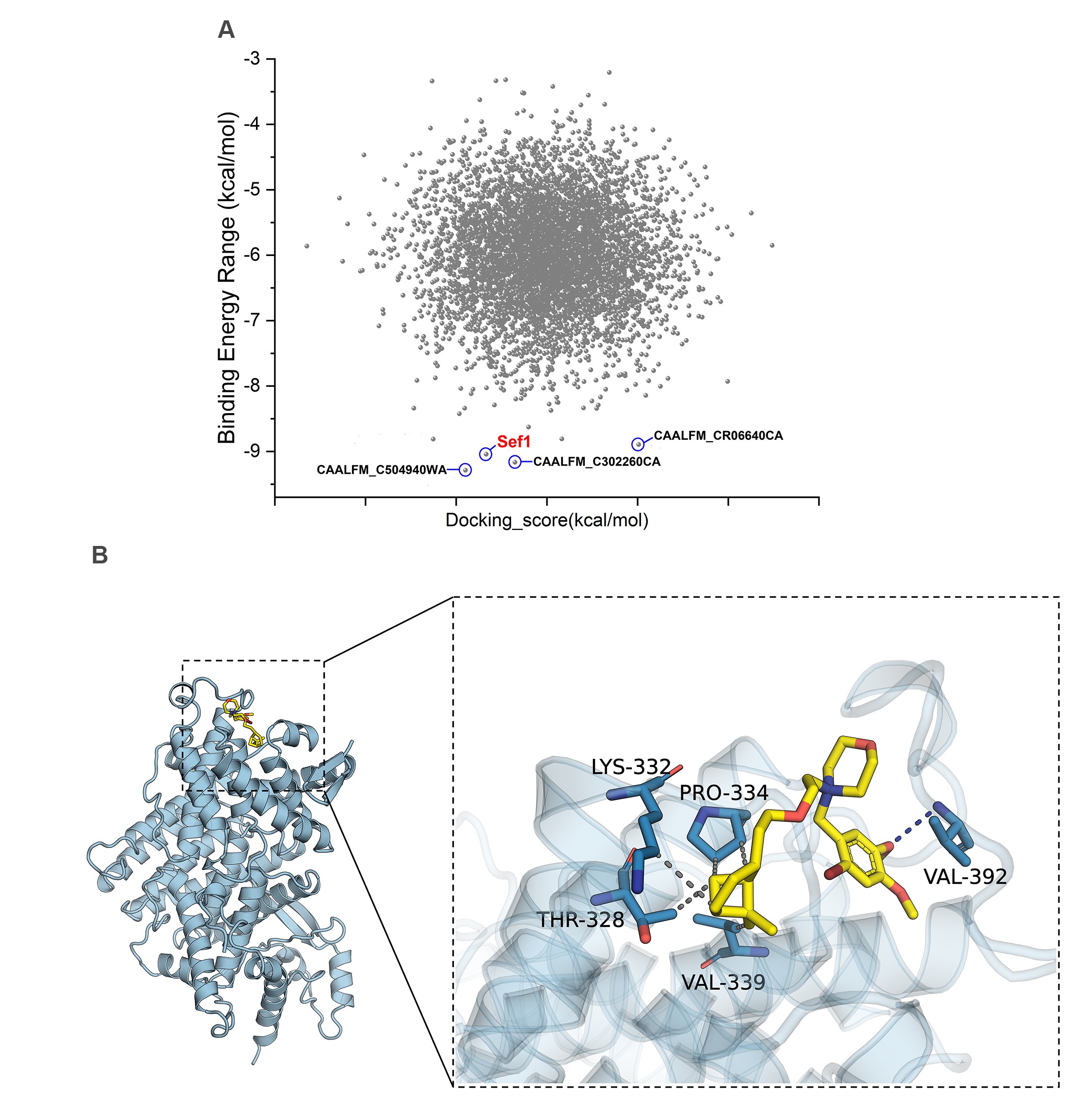


**Figure S5.** In silico analysis of the interaction between PB and Sef1. (A) Molecular docking score distribution for 5,973 proteins from *C. albicans* SC5314, obtained via batch molecular docking. (B) Predicted binding mode between PB and Sef1 derived from molecular docking. The left panel provides an overall view of the complex, while the right panel shows a detailed view of the interaction interface. Hydrogen bonds are indicated by blue lines, hydrophobic interactions by gray dashed lines, pi–pi stacking interactions by green dashed lines, and salt bridges by yellow dashed lines.

Supplementary reference

1. Gong Y, Li S, Wang W, et al. In vitro and in vivo activity of chelerythrine against *Candida albicans* and underlying mechanisms. Future Microbiol. 2019;14:1545-1557. doi: 10.2217/fmb-2019-0178.
