# Supplementary material for: Repurposing the antispasmodic drug pinaverium bromide as a novel antifungal agent and synergist against Candida albicans
Source: Virulence. 2026 Jun 1;17(1):2682573. doi: 10.1080/21505594.2026.2682573 (PMC13232878; doi:10.1080/21505594.2026.2682573)
Supplement: Ethics approval.pdf [file KVIR_A_2682573_SM8096.pdf]

动物实验伦理审查同意书
Affidavit of Approval of IACUC

申请编号 Application No.	14147	批准编号 Approval No.	IACUC-2106025

本《动物实验方案》经过实验动物管理与伦理委员会审核，符合动物保护、动物福利和伦理原则，符合国家动物福利伦理的相关规定。方案的相关信息如下

The animal use protocol listed below has been reviewed and approved by Institutional Animal Care and Use Committee (IACUC)

实验名称 Protocol Title	构建白色念珠菌感染的小鼠动物模型及药效评价<br>Establishment of an animal model with Candida albicans infection and Evaluating efficacy				
申请人姓名 Applicant	童倩<br>Tong Qian	职称/学位 Title/Degree	学士<br>bachelor	邮箱 Email	1033887352@qq .com
实验负责人 Principle Investigator	毕洪凯<br>Hongkai Bi	职称/学位 Title/Degree	教授/博士<br>professor/doctor	邮箱 Email	hkbi@njmu.edu .cn
院系（部门） Department	南京医科大学基础医学院<br>School of basic medicine, nanjing medical university			申请日期 Application Date	2021-05-30
拟实验时间 Period of Protocol	2021-07-01 - 2024-12-31	实验动物使用许可证 Number of Animal Use Permit		SYXK(苏)2020-0022	
审核意见 Results of Inspection	符合动物福利伦理要求，可以进行实验。Agree				
兽医师 Chief Veterinary Officer	马贯中<br>Ma Guanzhong	马贯中<br>Ma Guanzhong		日期 Date	2021.6.10

南京医科大学实验动物福利伦理委员会
Institutional Animal Care and Use Committee of NMU
主席(chairman)：施爱民　　**签名**(signature)：施爱民
Shi Aimin

日期(Date)：2021-06-10
